# Does *APOE* Genotype Modify the Relations Between Serum Lipid and Erythrocyte Omega-3 Fatty Acid Levels?

**DOI:** 10.1007/s12265-014-9554-8

**Published:** 2014-03-05

**Authors:** William S. Harris, James V. Pottala, Dawn L. Thiselton, Stephen A. Varvel, Alison M. Baedke, Thomas D. Dayspring, G. Russell Warnick, Joseph P. McConnell

**Affiliations:** 1 Health Diagnostic Laboratory, Inc., 737 N. 5th St, Suite 103, Richmond, VA 23219 USA; 2 Department of Internal Medicine, Sanford School of Medicine, University of South Dakota, Sioux Falls, SD USA; 3 Department of Psychiatry, Virginia Commonwealth University, Richmond, VA USA; 4 Foundation for Health Improvement and Technology, Richmond, VA USA

**Keywords:** *APOE* genotype, APOE4, Omega-3 fatty acids, Lipids, Low-density lipoproteins, Triglycerides, apoB

## Abstract

Earlier reports indicated that patients with the apolipoprotein *APOE* ε4 allele responded to fish oil supplementation with a rise in serum low-density lipoprotein cholesterol (LDL-C) compared to ε3 homozygotes. In this study, we used clinical laboratory data to test the hypothesis that the cross-sectional relation between RBC omega-3 fatty acid status (the Omega-3 Index) and LDL-C was modified by *APOE* genotype. Data from 136,701 patients were available to compare lipid biomarker levels across Omega-3 Index categories associated with heart disease risk in all *APOE* genotypes. We found no adverse interactions between *APOE* genotype and the Omega-3 Index for LDL-C, LDL particle number, apoB, HDL-C, or triglycerides. However, we did find evidence that ε2 homozygotes lack an association between omega-3 status and LDL-C, apoB, and LDL particle number. In summary, we found no evidence for a deleterious relationship between lipid biomarkers and the Omega-3 Index by *APOE* genotype.

**Electronic supplementary material:**

The online version of this article (doi:10.1007/s12265-014-9554-8) contains supplementary material, which is available to authorized users.

## Introduction

Apolipoprotein E (APOE) is found in the triglyceride-rich lipoproteins, i.e., very low-density lipoprotein (VLDL) particles, chylomicrons, VLDL and chylomicron remnant lipoproteins, intermediate density lipoproteins, and some very large high-density lipoproteins (HDL). APOE is a ligand, along with apoB, for the LDL (or apoB/E) receptor as well as the LDL receptor-related protein and the VLDL receptor, and mediates aspects of lipoprotein clearance, lipidation, and delipidation [1]. The *APOE* gene is polymorphic, that is, it can exist in three different forms, with alleles ε2, ε3, and ε4 coding for apoprotein isoforms APOE2, APOE3, and APOE4. Any given patient will carry up to two different isoforms, and ethnic variations in genotype frequency have been reported [2]. Differences in APOE isoforms affect their affinity for the LDL and VLDL receptors and the apoC content of VLDL, thus affecting lipolytic conversion of VLDL to LDL particles. The lipid phenotypic expression of different genotypes varies depending on environmental stressors (e.g., smoking, diet), comorbidities (e.g., obesity, diabetes), and other genetic factors [3, 4]. Patients who are ε3/ε4 or ε4/ε4 have elevated LDL levels and are at increased risk for coronary heart disease (CHD) compared to those with other phenotypes [5]. Accordingly, *APOE* genotyping can provide information regarding the causes of some dyslipidemias and, ultimately, risk for CHD. Knowledge of *APOE* genotype can also help guide therapy as certain drugs [3] and dietary patterns [6] affect lipid levels differently according to genotype.

An intervention for which *APOE* genotype may influence the lipid response is the use of fish oil (omega-3 fatty acids; eicosapentaenoic acid (EPA) and docosahexaenoic acid (DHA)) [7–11]. One report concluded, “In APOE4 carriers, the hypotriglyceridemic benefits [of fish oil] may be counteracted by a potential proatherogenic shift in the cholesterol profile” [7], and another stated, “High dose DHA supplementation is associated with increases in total cholesterol in E4 carriers, which appears to be due to an increase in LDL-C and may in part negate the cardioprotective action of DHA in this population subgroup taking omega-3” [8]. Although a statistically significant interaction between *APOE* genotype and fish oil on the low-density lipoprotein cholesterol (LDL-C) response has been reported [8], a potentially important question remains: are APOE4 carriers more likely to experience a rise in LDL-C with fish oil treatment than noncarriers? This question was also recently addressed in an intervention study by Thifalt et al. [12] and cross-sectionally in the Multi-Ethnic Study of Atherosclerosis (MESA) [12]. In neither case did genotype influence the relationship between omega-3 fatty acids and LDL-C.

The purpose of this study was to explore this question using a large clinical database in which we tested the hypothesis that the *APOE* genotype modulates the relationship between blood omega-3 fatty acid levels and serum LDL-C. In addition to LDL-C, we also examined the relations between omega-3 fatty acid status and a variety of other lipid markers [high-density lipoprotein (HDL)-C, triglycerides, LDL particle number (P), apolipoprotein B-100 (apoB)]. The biomarker of omega-3 fatty acid status used was the Omega-3 Index, i.e., the red blood cell (RBC) level of EPA + DHA [13–15].

## Methods

### Patients

All samples processed between July 2011 and April 2012 at Health Diagnostic Laboratory, Inc. (Richmond, VA) with the following data available—age, gender, *APOE* genotype, Omega-3 Index, and lipids/lipoproteins—were included in the cross-sectional analysis. The use of de-identified patient data for this analysis was approved by the Copernicus Group IRB (Durham, NC).

### Laboratory Methods

RBC fatty acid composition was analyzed according to the HS-Omega-3 Index® methodology as modified from Harris et al. [15]. Fatty acid methyl esters were generated from erythrocytes by transesterification with boron trifluoride and analyzed by gas chromatography. Fatty acids were identified by comparison with a standard mixture of fatty acids characteristic of RBCs. Omega-3 Index is given as EPA plus DHA expressed as a percentage of total identified fatty acids after response factor correction (based on calibration curves). *APOE* genotyping was performed by TaqMan assay (Applied Biosystems Inc., Carlsbad, CA) with a success rate of >95 %. The ε2, ε3, and ε4 single nucleotide polymorphisms detected were on chromosome 19 at positions rs429358 and rs7412 with the following thymidine (T) and cytosine (C) residues in the first position of the codon: TT, TC, and CC, respectively. Triglyceride assay was performed using standard automated enzymatic methods on a Roche/Hitachi P-Modular system with Roche reagents (Roche Diagnostics, Indianapolis, IN). ApoB was analyzed using an immunoturbidimetric assay from Roche Diagnostics on a Roche/Hitachi P-Modular system. LDL-C and HDL-C were measured using direct enzymatic assays from Randox (County Antrim, UK) on a Roche/Hitachi P-Modular system. Low-density lipoprotein particle (LDL-P) was measured at LipoScience (Raleigh, NC) by nuclear magnetic resonance technology as described previously [16].

### Statistical Methods

Differences in lipids among the *APOE* genotypes were tested using one-way ANOVA, and multiple testing compared to the ε3/ε3 group was controlled using Dunnett adjusted *p* values <0.05 for statistical significance. The lipids were transformed using natural logarithm to improve normality and homoscedasticity of the residuals; hence, geometric means (95 % CI) were reported.

Next, the lipid/lipoprotein biomarkers (i.e., LDL-C, LDL-P, apoB, HDL-C, triglycerides) were the dependent variables in linear models adjusted for age and gender. To test the hypothesis that the relations between lipid endpoints and the Omega-3 Index were modified by *APOE* genotype, the Omega-3 Index, *APOE* genotype, and their interaction term were also included in all models. The Omega-3 Index was categorized as (<4, 4–8, and >8 %) corresponding to three different cardiovascular disease (CVD) risk levels (high, intermediate, and low, respectively) [13] and was alternatively included in the model as a continuous variable for a sensitivity analysis. To determine how the *APOE* genotype should be included in the model, the following genetic inheritance models were tested: additive, dominant, recessive, and codominant. The estimated genotype association parameters were exponentiated and reported as relative percent differences in endpoints. Analyses were performed using SAS® software (version 9.3; SAS Institute).

## Results

**Fig. 1:**
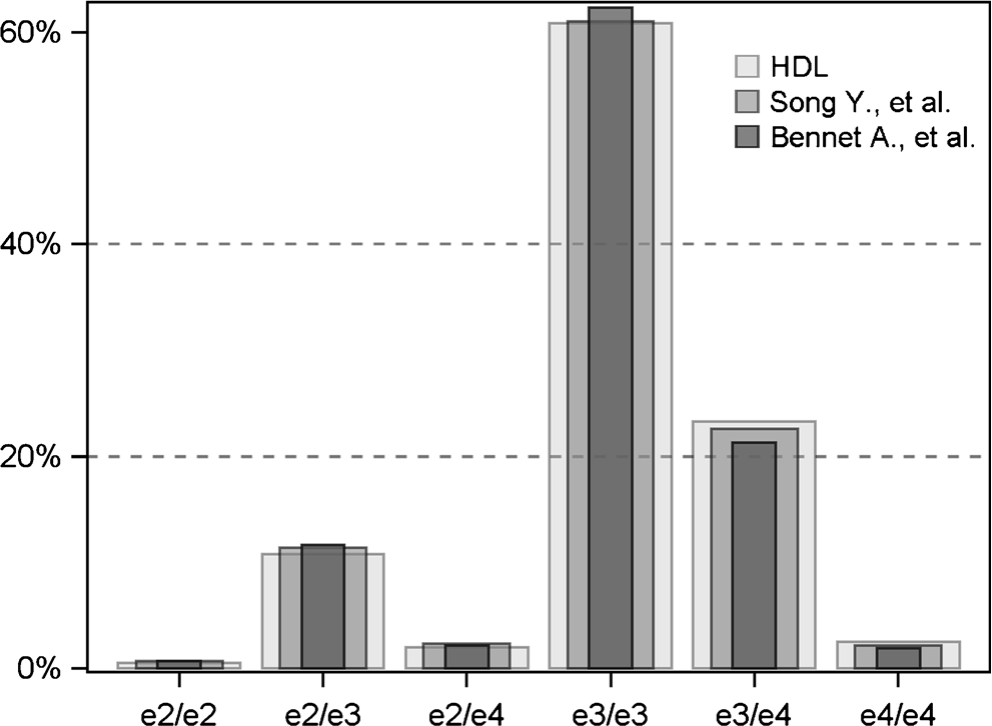
Distribution of apolipoprotein E (*APOE*) genotypes in the cohort (*N* = 136,701) compared to those reported from two meta-analyses (Song et al. [5] and Bennet et al. [17])

The mean (SD) age in this cohort (*N* = 136,701) was 57 (15) years, and 48 % of the samples were from males. The distribution of *APOE* genotype is shown in Fig. 1. There was a direct association between the genotype rank order (i.e., ε2/ε2, ε2/ε3, ε2/ε4, ε3/ε3, ε3/ε4, ε4/ε4) and plasma levels of LDL-C (and other atherogenic lipoprotein markers such as apoB and LDL-P; Table 1). That is, ε2/ε2 patients had the lowest levels and ε4/ε4 the highest. There were small differences in HDL-C and the Omega-3 Index by genotype. The ε2/ε2 group had an elevated geometric mean triglyceride level that was 36 mg/dL greater than the ε3 homozygote reference group.

**Table 1:** Geometric mean (95 % CI) of lipid biomarkers by *APOE* genotype (*N* = 136,701)

Variable	ε2/ε2	ε2/ε3	ε2/ε4	ε3/ε3 (ref)	ε3/ε4	ε4/ε4
	*n* = 860	*n* = 15,377	*n* = 3,098	*n* = 82,467	*n* = 31,673	*n* = 3,226
Omega-3 Index [% total fatty acids]	4.62 (4.51, 4.74)	4.61^a^ (4.59, 4.64)	4.54^a^ (4.48, 4.59)	4.65 (4.64, 4.67)	4.59^a^ (4.57, 4.61)	4.60 (4.54, 4.65)
HDL-cholesterol [mg/dL]	50.7 (49.7, 51.7)	52.0^a^ (51.8, 52.2)	51.5 (51.0, 52.0)	51.5 (51.3, 51.5)	50.6^a^ (50.4, 50.7)	50.8 (50.4, 51.3)
Triglycerides [mg/dL]	149^a^ (143, 156)	120^a^ (119, 121)	119^a^ (117, 122)	113 (113, 114)	115^a^ (114, 116)	116^a^ (114, 119)
LDL-cholesterol [mg/dL]	45.2^a^ (44.2, 46.3)	79.9^a^ (79.4, 80.3)	85.7^a^ (84.7, 86.7)	94.6 (94.4, 94.8)	98.9^a^ (98.6, 99.3)	102^a^ (101, 104)
ApoB [mg/dL]	51.7^a^ (50.6, 52.9)	76.2^a^ (75.8, 76.6)	80.9^a^ (80.0, 81.8)	86.2 (86.1, 86.4)	90.2^a^ (89.9, 90.5)	93.8^a^ (92.9, 94.7)
LDL-particle [nmol/L]	694^a^ (675, 715)	1,208^a^ (1,201, 1,216)	1,295^a^ (1,277, 1,313)	1,435 (1,431, 1,438)	1,504^a^ (1,498, 1,510)	1,552^a^ (1,531, 1,572)

^a^Genotype differences were tested using one-way ANOVA, and multiple testing compared to the ε3/ε3 group was controlled using Dunnett adjusted *p* value <0.05

**Fig. 2:**
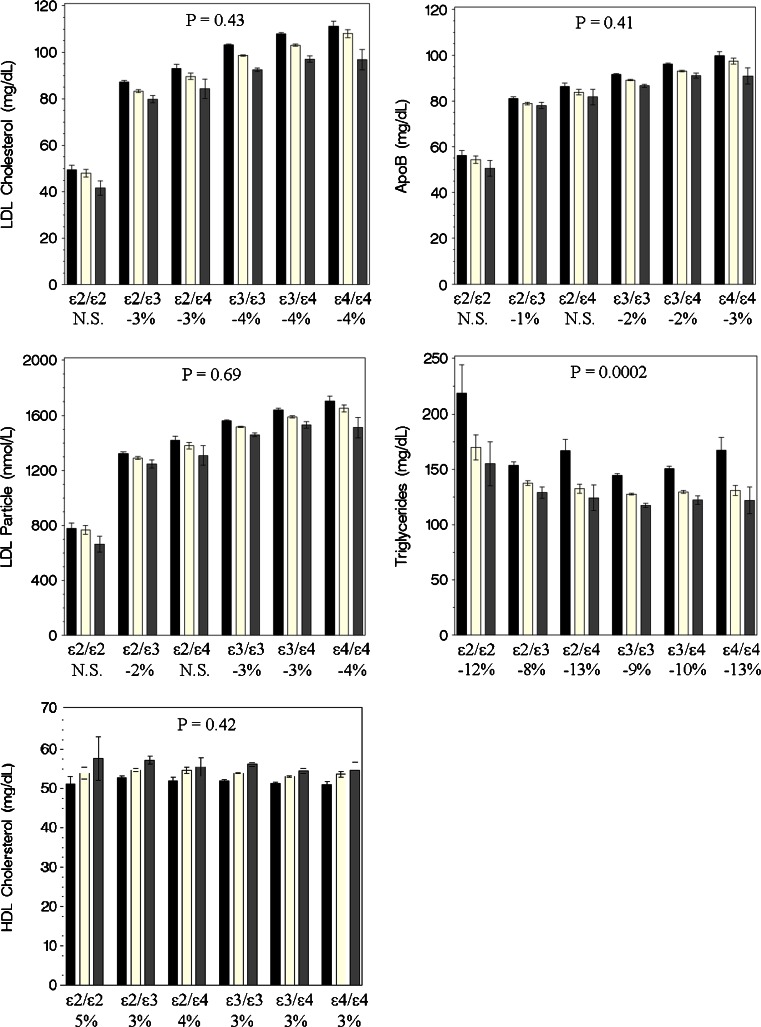
Mean (95 % CI) for lipid/lipoprotein biomarkers by *APOE* genotype shown across CHD risk categories of the Omega-3 Index (RBC EPA + DHA) (*black bars* <4 %, *white bars* 4 to 8 %, *gray bars* >8 %). *p* values for ordinal slope interactions (adjusted for age and gender) between *APOE* genotype and Omega-3 Index levels are given in panels. Slope (% change) between each level of the Omega-3 Index within genotype was significant (*p* ≤ 0.0083, 0.05 / 6 genotypes) with estimates shown below genotypes, unless noted as not significant (N.S.). *N* = 136,701

The genetic inheritance model revealed that the ε4 allele’s association with LDL-C was additive and also that the ε2 allele was codominant, meaning that homozygotes had a diverse nonadditive association with LDL-C compared to heterozygotes (Table 1 and Supplemental Table 1). Due to the complex relations with LDL-C and the large sample size, all six *APOE* genotypes were reported throughout. The cross-sectional relations of lipid/lipoprotein biomarkers with the Omega-3 Index CVD risk groups are shown by genotype in Fig. 2. A decrease in mean atherogenic marker levels with increasing Omega-3 Index categories was observed across all genotypes with significant ordinal slopes (*p* ≤ 0.0083, i.e., 0.05 / 6 genotypes). The only exceptions were for LDL-C, LDL-P, and apoB in the ε2/ε2 patients, and for LDL-P and apoB in the ε2/ε4 group. HDL-C means had significant positive slopes with increasing Omega-3 Index levels. Importantly, there were no significant genotype interactions across Omega-3 Index CVD risk groups for LDL-C, apoB, LDL-P, and HDL-C in age- and gender-adjusted linear models (min. interaction *p* = 0.41, Fig. 2, Table 2). However, the relations between the Omega-3 Index and triglycerides were modified by *APOE* genotype, varying from a −8 % to a −13 % decrease per category increase in the Omega-3 Index (interaction *p* = 0.0002).

**Table 2:** Omega-3 Index and *APOE* genotype interaction *p* values

*N* = 136,701 endpoint	Omega-3 Index ordinal (Fig. 3)	Omega-3 Index continuous
Ln(LDL-C)	0.43	0.018^a^
Ln(LDL-P)	0.69	0.067
Ln(ApoB)	0.41	0.011^a^
Ln(HDL-C)	0.42	0.59
Ln(triglycerides)	0.0002	0.0004^a^

Models were adjusted for age and gender and included *APOE* genotype as a categorical variable, the Omega-3 Index (as either ordinal or continuous), and their interaction

^a^See Table 3 for more detailed information

A more sensitive analysis examined the Omega-3 Index as a continuous variable and detected an interaction with *APOE* genotype on LDL-C, apoB, and triglycerides (Table 2). The *APOE* genotype modified the association between these lipids and a 1 % (of total RBC fatty acids) increase in the Omega-3 Index. The interaction with *APOE* genotype and the Omega-3 Index on LDL-C was significant, not due to a unique association in the ε4 subjects but due to a lack of association in the ε2/ε2 group (−0.3 %, *p* = 0.57) and a significant attenuation in the ε2/ε3 group compared to the ε3 homozygotes (−0.8 vs. −1.3, *p* = 0.0023) (Table 3). Similarly, the interaction detected for apoB was also due to the ε2 homozygotes’ nonassociation and the ε2 heterzygotes’ attenuated association compared to the other genotypes. Overall, there were no adverse relationships between higher omega-3 levels and lipid biomarker levels regardless of the *APOE* genotype.

**Table 3:** Cross-sectional percent differences (95 % CI) in lipids associated with a 1 % higher Omega-3 Index by *APOE* genotype (*N* = 136,701)

Endpoint	ε2/ε2	ε2/ε3	ε2/ε4	ε3/ε3	ε3/ε4	ε4/ε4
	*n* = 860	*n* = 15,377	*n* = 3,098	*n* = 82,467	*n* = 31,673	*n* = 3,226
Ln(LDL-C)	−0.3 (−1.4, 0.8) *p* = 0.57	−0.8 (−1.1, −0.6) *p* < 0.0001	−1.0 (−1.6, −0.4) *p* = 0.0014	−1.3 (−1.4, −1.2) *p* < 0.0001	−1.3 (−1.5, −1.1) *p* < 0.0001	−1.5 (−2.1, −0.8) *p* < 0.0001
Ln(ApoB)	0.0 (−0.7, 1.2) *p* = 0.62	−0.2 (−0.5, 0.0) *p* = 0.042	−0.5 (−1.1, 0.0) *p* = 0.065	−0.6 (−0.7, −0.5) *p* < 0.0001	−0.7 (−0.9, −0.5) *p* < 0.0001	−1.0 (−1.5, −0.4) *p* = 0.0003
Ln(triglycerides)	−2.9 (−4.6, −1.1) *p* = 0.0020	−2.9 (−3.3, −2.4) *p* < 0.0001	−4.1 (−5.1, −3.1) *p* < 0.0001	−3.1 (−3.3, −3.0) *p* < 0.0001	−3.7 (−4.0, −3.4) *p* < 0.0001	−4.5 (−5.4, −3.5) *p* < 0.0001

The interactions between the Omega-3 Index and *APOE* genotype seen in Table 2 were due to ε2/ε2 and ε2/ε3 patients having attenuated associations between the Omega-3 Index and lipid endpoints

## Discussion

The basic question that prompted this research project was, “are APOE4 carriers more likely to experience a rise in LDL-C with fish oil treatment than noncarriers?” To address this question cross-sectionally, we asked if the relationships between a marker of omega-3 fatty acid intake (the Omega-3 Index) and several lipid biomarkers differed by *APOE* genotype.

We explored this question using a large dataset of *APOE*-genotyped patients being tested for cardiovascular risk markers. An important question is how well this clinical dataset reflects the general population. Accordingly, we compared our *APOE* genotype distribution with those reported in two meta-analyses, one from over 45,000 individuals in 48 separate studies worldwide [5] and the other from 22 studies including over 70,000 subjects [17] (Fig. 1). Distributions were very similar. Further indicators of representativeness were the previously reported direct associations of atherogenic lipid markers by genotype rank order (i.e., from ε2/ε2 through ε4/ε4) [17, 18] (Table 1). Together, these observations suggest that this clinical cohort was reasonably representative of other populations, and therefore, the associations observed here are likely to be generalizable.

To address the primary research hypothesis, we tested the cross-sectional associations between three different ranges of the Omega-3 Index (representing different CHD risk categories) and serum lipid/lipoprotein markers across genotypes (Fig. 2); as a sensitivity analysis, we replaced the Omega-3 Index CVD risk categories with the continuous measure. If lipid biomarkers changed with increasing omega-3 status in some genotypes but not in others, then that would stand as evidence that genotype *does* modulate the relations between omega-3 fatty acid levels and these risk markers. We found no interactions between the Omega-3 Index risk categories and *APOE* genotype on LDL-C, LDL particle number, apoB, or HDL-C. However, the relationship between the Index and triglycerides did depend on genotype; compared to the reference group (ε3/ε3), the decrease in triglycerides associated with increasing Omega-3 Index CVD risk categories ranged from −8 % for ε2/ε3 to −13 % for ε2/ε4 and ε4/ε4. When the Omega-3 Index categories were replaced with the continuous measure, two additional *APOE* interactions were detected. These revealed a lack of association between the Omega-3 Index with LDL-C or apoB in the ε2 homozygotes, and an attenuated association in the ε2 heterozygotes. To the point of this paper, there were no unique relationships between atherogenic lipids and omega-3 status associated with the ε4 genotype. In addition, none of the lipid biomarkers had an adverse cross-sectional relation with increasing Omega-3 Index levels. This confirms the favorable relations between cardiovascular risk profile and the Omega-3 Index recently reported in Framingham [19].

The MESA investigators also examined the question of the relations between omega-3 fatty acid status, lipid biomarkers, and *APOE* genotype [12]. There were several differences between their study and ours that make a comparison of results difficult. Our study included 136,701 (vs. 2,340) patients, had minimal (vs. substantial) information on covariates, measured RBC membrane (vs. plasma phospholipid) fatty acids, used a combined metric of EPA + DHA (vs. each alone), and tested different relations with lipid/lipoprotein endpoints. Importantly, both studies reported no *APOE* gene-fatty acid adverse interactions for LDL-C or LDL-P. However, the relations between EPA levels and these biomarkers were direct in the report of Liang et al. In MESA, there was a significant interaction by genotype (ε2) between EPA and HDL-C (direct relations between these markers in the ε2 patients but not in the other genotypes), whereas our study showed direct relations in all *APOE* genotypes. Both studies found that omega-3 fatty acid levels were slightly lower in the ε4 and ε2 carriers (compared to the ε3 homozygotes); however, the mean differences were small (about 0.05 %) and of doubtful clinical significance.

Although our primary focus was on the ε4 allele, the ε2/ε2 genotype may be the more unique genotype with respect to omega-3 fatty acids. In this group (*n* = 860), there was no significant association between the Omega-3 Index and LDL-C, apoB, and LDL-P. The ε2/ε2 patients had by far the lowest baseline LDL-C, apoB, and LDL-P concentrations, so a lack of association perhaps has little clinical relevance.

The effects of omega-3 treatment on lipid profiles in patients carrying an ε4 allele was examined in several previous studies, most from the University of Reading, UK. In the first, despite finding no significant effect of genotype on serum lipid responses to fish oil, the authors stated that, “In APOE4 carriers, the hypotriglyceridemic benefits [of fish oil] may be counteracted by a potential proatherogenic shift in the cholesterol profile” [7]. This conclusion was widely construed by many clinicians to mean that APOE4 carriers should not be given fish oil. A follow-up study reported no effect of genotype, but the dose of omega-3 fatty acids was rather low [9]. In the third study [8], normal volunteers (*n* = 20 for ε3/ε3 vs. *n* = 18 for ε3/ε4) were given either 3.7 g/day of DHA or 3.3 g/day of EPA. The latter had no effect on LDL-C in either group, but the former increased LDL-C by 10 % from baseline in the ε4 carriers compared with a 4 % decrease in the reference group. Of note, apoB was not differentially affected by genotype nor was LDL-P raised by DHA [20]. A 2012 study including 88 subjects failed to confirm the adverse DHA effect [10]. There was no *APOE* genotype interaction for postprandial lipid responses while on a fish oil diet [21] nor did the authors find a genotype interaction for the effect of fish oil on LDL-C. Our findings, and those from the MESA investigators [12], are consistent with the majority view that the relationship between omega-3 fatty acid blood levels and LDL-C is not different in ε4 carriers relative to that in the common wild-type genotype.

Taking a wider view, it is well known that ε4 patients are at higher risk for CHD [5, 17] and for Alzheimer’s disease [22]. Even if the efficacy of fish oil supplements as treatments for CHD is in question [23, 24], several studies have shown that higher omega-3 fatty acid blood levels are associated with decreased risk for all-cause mortality [25–28] and dementia [29]. Direct consumption of EPA and DHA (whether from fish oil supplements or oily fish) is by far the most important determinant of the Omega-3 Index [30–33]. Their safety profile is strong [34], and thus, their risk/benefit ratio is favorable. Finally, because ε4 carriers may require higher doses of EPA + DHA to raise the Omega-3 Index (as suggested in some [35, 36] but not other [10] studies), patients carrying this allele may be the most likely to benefit from an increased omega-3 fatty acid intake.

## Strengths and Limitations

Strengths of the study include a very large sample size, the use of an objective biomarker sensitive to changes in omega-3 fatty acid intake (the Omega-3 Index), and a broad spectrum of unselected patients with an *APOE* genotype distribution similar to that in other cohorts. There were also limitations; this was not a randomized trial of fish oil supplementation in patients with different *APOE* genotypes but a retrospective, medical records-based analysis. As such, we had no data on comorbidities or lifestyle factors for these patients. Perhaps more importantly, we had no information on concomitant drugs, and it is possible that pharmacologic regimens differed by genotype. This could confound the relations observed here and limit the conclusions that could be drawn from them.

## Electronic Supplementary Material

Below is the link to the electronic supplementary material.

ESM 1 (DOCX 11 kb)
