# Supplementary material for: Does APOE Genotype Modify the Relations Between Serum Lipid and Erythrocyte Omega-3 Fatty Acid Levels?
Source: J Cardiovasc Transl Res. 2014 Mar 5;7(5):526–32. doi: 10.1007/s12265-014-9554-8 (PMC4098055; doi:10.1007/s12265-014-9554-8)
Supplement: Supplementary file 1 — (DOCX 11 kb) [file 12265_2014_9554_MOESM1_ESM.docx]

**Supplemental Table 1. LDL-C one-way ANOVA Inheritance Models**

| **ε4 allele**  **Inheritance Model**  **N = 120,464** | **AIC** | **ε2 allele**  **Inheritance Model**  **N = 101,802** | **AIC** |
| --- | --- | --- | --- |
| Co-dominant | 77850 | ***Co-dominant*** | ***65560*** |
| ***Additive*** | ***77846*** | Additive | 66705 |
| Dominant | 77898 | Dominant | 68011 |
| Recessive | 78063 | Recessive | 68836 |

**ε**3 was the wild type; AIC = Akaike’s Information Criterion;

The best fitting model (i.e. minimum AIC) is shown in ***bold, italic***.
